# Comparative evaluation of the hygienic efficacy of an ultra-rapid hand dryer *vs* conventional warm air hand dryers

**DOI:** 10.1111/j.1365-2672.2010.04838.x

**Published:** 2011-01

**Authors:** AM Snelling, T Saville, D Stevens, CB Beggs

**Affiliations:** 1 Bradford Infection Group, University of BradfordBradford, West Yorkshire, UK; 2 Microbiology Department, Dyson LimitedTetbury Hill, Malmesbury, Wiltshire, UK

**Keywords:** contamination, cross-contamination, hand drying, hand hygiene, hygiene, skin microflora

## Abstract

**Aims:**

To compare an ultra-rapid hand dryer against warm air dryers, with regard to: (A) bacterial transfer after drying and (B) the impact on bacterial numbers of rubbing hands during dryer use.

**Methods and Results:**

The Airblade™ dryer (Dyson Ltd) uses two air ‘knives’ to strip water from still hands, whereas conventional dryers use warm air to evaporate moisture whilst hands are rubbed together. These approaches were compared using 14 volunteers; the Airblade™ and two types of warm air dryer. In study (A), hands were contaminated by handling meat and then washed in a standardized manner. After dryer use, fingers were pressed onto foil and transfer of residual bacteria enumerated. Transfers of 0–10^7^ CFU per five fingers were observed. For a drying time of 10 s, the Airblade™ led to significantly less bacterial transfer than the other dryers (*P*<0·05; range 0·0003–0·0015). When the latter were used for 30–35 s, the trend was for the Airblade to still perform better, but differences were not significant (*P*>0·05, range 0·1317–0·4099). In study (B), drying was performed ± hand rubbing. Contact plates enumerated bacteria transferred from palms, fingers and fingertips before and after drying. When keeping hands still, there was no statistical difference between dryers, and reduction in the numbers released was almost as high as with paper towels. Rubbing when using the warm air dryers inhibited an overall reduction in bacterial numbers on the skin (*P* < 0·05).

**Conclusions:**

Effective hand drying is important for reducing transfer of commensals or remaining contaminants to surfaces. Rubbing hands during warm air drying can counteract the reduction in bacterial numbers accrued during handwashing.

**Significance and Impact of the Study:**

The Airblade™ was superior to the warm air dryers for reducing bacterial transfer. Its short, 10 s drying time should encourage greater compliance with hand drying and thus help reduce the spread of infectious agents via hands.

## Introduction

Handwashing is a hugely important infection control measure in clinical, manufacturing and domestic environments. A great deal of research has focussed on such aspects as handwashing technique, efficacy of antimicrobial handwash agents, how to improve compliance and the effect of wearing jewellery (Pittet 2000; Jumaa 2005; Pittet *et al.* 2006; Rotter *et al.* 2009). In contrast, comparatively little research has been carried out to quantify the contribution that hand drying makes to the overall effectiveness of the washing event. With most handwashing regimens, the numbers of bacteria on the skin surface are lowered, but not eliminated. If hands are not then dried properly, transfer of commensal strains, or transients not eliminated by the wash itself, is more likely to occur (Gould 1994; Merry *et al.* 2001). The degree of wetness of hands appears to greatly influence bacterial transfer and dissemination to surfaces and items touched. This probably occurs not only because of the physical aspects of moisture droplets transferring between one surface and another but also because the bacteria may be maintained in a physiological state that makes them better able to survive in the new environment. Patrick *et al.* (1997) reported that by drying the hands, the numbers of bacteria transferred to samples of skin, food or utilities were reduced by an order of 99%.

If hands repeatedly remain damp because of ineffective hand drying, it can lead to skin excoriation which in turn can lead to altered and higher populations of bacteria colonizing the skin. This has been found to be a particular problem amongst certain cohorts of nurses, where routine duties require multiple instances of hand washing per hour. It can lead to greater carriage of Gram-negative bacteria plus yeasts. More worryingly, *Staphylococcus aureus* can become established as part of their normal skin flora (Larson *et al.* 1998). Hand drying to decrease microbial counts at the skin surface is now recognized as an essential part of handwashing procedures aimed at reducing the spread of methicillin-resistant *Staph. aureus* (MRSA) in hospitals (Collins and Hampton 2005), but few protocols stipulate exactly how to dry the hands, or for how long. Even EN1499:1997 (BSI 1997), the European Standard Handwash technique widely used in laboratory and field studies, does not incorporate a precisely defined hand drying step, prior to microbiological sampling.

The four main methods of hand drying are letting the skin dry by evaporation, use of paper towels, cloth towels, or, in more recent times, use of warm air dryers. Whilst studies have found warm air dryers to be equivalent (Taylor *et al.* 2000) or even superior (Ansari *et al.* 1991) to paper towels for reducing numbers of micro-organisms on the hands, concerns have been raised about their overall hygiene. There have been conflicting reports regarding dispersal of bacteria in the washroom environment via aerosols liberated from the machines (Matthews and Newsom 1987; Redway 1994; Redway and Knight 1998; Taylor *et al.* 2000). Another issue concerns the need to rub the hands vigorously under the warm air stream, because this can cause increased bacterial counts on the skin surface after washing (Yamamoto *et al.* 2005).

**Figure 1:**
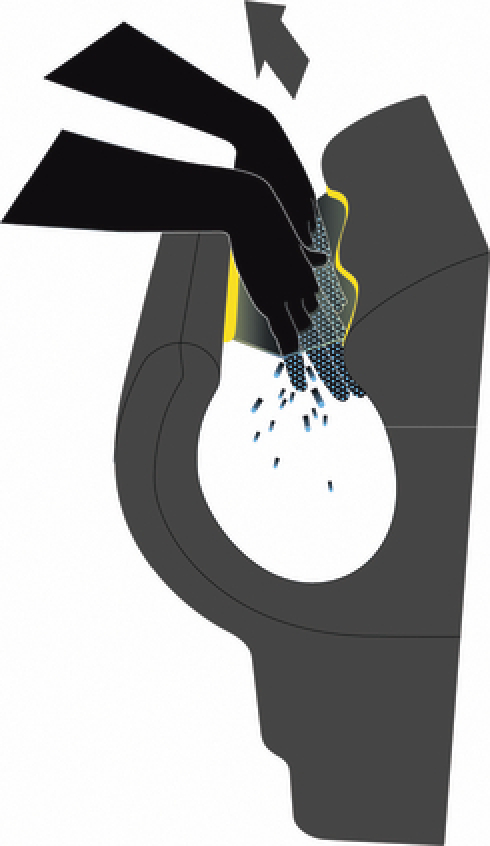
Simplified view of moisture being ‘stripped’ from the hands by the air knives of the Dyson Airblade™ hand dryer.

The Airblade™ (Dyson Ltd, Malmesbury, UK) is a new type of dryer that aims to address some of the problems outlined earlier. Air is drawn in through a HEPA filter at the base of the machine, through the motor and expelled through two 0·3 mm-wide slits, creating two high pressure ‘knives’ of filtered air (Fig. 1). The hands are inserted into the cavity between the slits, and sensors start the airflow automatically. The hands are then drawn up slowly through the sheet of air generated, and water on the hands is stripped off in a controlled manner. This process does not rely on evaporation of moisture from the skin, so the air does not need to be heated and the hands do not need to be rubbed to speed the process. The water that is removed is deposited onto the fascia of the machine, which uses a hydrophilic coating to spread the water out facilitating evaporation in the turbulent airflow. As the hands are held apart and drawn upwards through the airstream, drying takes just 10–12 s.

In this study, the Airblade™ was trialled against two models of conventional warm air dryers, typical of those widely used in public washrooms. In study (A), the impact of the hand drying process on the transfer of bacteria from fingers to an inert surface was quantified. In Study (B), the effect of hand rubbing was assessed and compared to the process of drying the hands with paper towels. The results provide an evidence base for the development and enhancement of hygienic hand drying practices.

## Materials and methods

### Volunteers

Fourteen volunteers (seven men and seven women) were recruited to take part in the study. All were over 18 years of age and gave written informed consent for their participation. Because of the need to handle raw meat during the tests, anyone with open sores or abrasions on either hand was excluded from participating. Volunteers were not currently receiving treatment for dermatological disorders and had not used oral or topical antibiotics during the previous 3 months.

### Hand dryers

Two models of warm air hand dryer were used: a manual operation A5 (World Dryer Corporation, Berkeley, IL, USA) and an automatic operation Turbodry™ (PHS Group plc, Caerphilly, UK). These are typical of dryers found in washrooms around the world, whereby unfiltered air is warmed and blown vertically downwards whilst hands are rubbed together in the airstream. Upon activation, the A5 unit stays on for 30 s and the Turbodry™ for 35 s. A Dyson Airblade™ dryer (240v UK model; Dyson Ltd) was also tested. This is an ultra-rapid dryer that uses two high-pressure ‘knives’ of HEPA-filtered air (at ambient temperature) to strip water from hands that are held apart as they are drawn upwards through the airstream. The manufacturers recommended time for drying hands with this machine is 10 s.

To avoid the risk of any pre-existing internal microbial contamination seeding the outlet air streams, new models of each type of dryer were used.

### Study (A): quantification of bacteria transferred to an inert surface from the fingertips after drying

First, the hands of volunteers were deliberately contaminated by manipulation of a fresh, uncooked chicken, washed in the manner described below and then dried using a variety of methods. After drying, the volunteers touched strips of aluminium foil. Any bacteria transferred to the foil from the fingertips were then enumerated by standard culture techniques. In this way, transfer to an inert surface of any bacterial contaminants that survived the washing plus drying steps was quantified.

#### Standardized contamination and hand washing procedure

For all tests in this study, volunteers contaminated their hands (both left and right) by manipulating a fresh, uncooked chicken, as recommended by Charbonneau *et al.* (2000). Chickens were purchased from a local supermarket, stored at 4°C and used within 48 h. One chicken was used per volunteer per day. To contaminate the hands, the chicken was massaged inside and out for 45 s, then hands were allowed to air-dry for 1 min. Following this, hands were washed in tap water at a temperature of 40 ± 5°C and a constant flow rate of 4 l min^−1^. For each handwash, a single squirt (1·5–2 g) of nonmedicated liquid soap (Sommerfield Jasmin luxury cream handwash) was dispensed into the palm of the right hand. The hands were then washed for a full 60 s in accordance with the actions described in EN1499:1997 (BSI 1997).

#### Hand drying procedures

The majority of the tests in Study (A) used a standard drying time of 10 s for all hand dryers. This drying time was selected because it is the time recommended for the Airblade™ machine. As a control, some tests involved allowing hands to dry naturally in the room air, without movement or rubbing of any kind. In addition, experimental runs utilizing the A5, and Turbodry™ machines were repeated using their activation period as the drying time.

On four separate occasions (different days or am and pm of the same day), each volunteer reported to the study laboratory, where they contaminated and washed their hands in the manner described earlier. The hands were then dried using one of the three dryers, or alternatively, allowed to dry naturally in the room air, as determined by a randomization table. When using the conventional warm air dryers, the volunteers rubbed their hands in their normal manner. With the Airblade™, hands were held still. When no dryer was to be used, they held their hands still for 10 s, with fingers pointing upwards and spread out.

#### Quantification of bacteria transferred to an inert surface from the fingertips

Immediately after each drying event, the amount of bacteria transferred from the fingertips when touching aluminium foil was enumerated, with transfer from the left and right hands being quantified separately. This gave a total of 28 replicates (i.e. 14 volunteers × 2 hands) per drying method.

Volunteers pressed each finger (one at a time) onto a strip (*c.* 10 × 4 cm) of sterile aluminium foil. One strip was used per five fingers of each hand. As each finger made contact with the foil, it was gently rolled, as if being fingerprinted. After being sampled, volunteers washed their hands thoroughly with an antiseptic handwash to remove any remaining contamination acquired from the chickens.

Using aseptic technique, each foil strip was curled loosely and deposited into a universal bottle containing 5 ml of Maximum Recovery Diluent (MRD; Oxoid, Basingstoke, UK) with 2% Tween 80 (Sigma-Aldrich, Poole, Dorset, UK), 0·1% lecithin and 0·1% sodium thiosulphate to neutralize any soap residue (Leyden *et al.* 1991). The bottles were then vortexed for *c.*20 s to resuspend any bacteria adhering to the foil. Serial tenfold dilutions were then made in MRD, and 100-μl aliquots of each dilution (plus neat) was plated onto the surface of duplicate plates of tryptone soya agar (TSA). Plates were incubated overnight at 37°C, and the resulting colonies counted. Results were recorded as colony forming units (CFU) per right or left fingertips (i.e. average count from the 5 ml of diluent used for each hand).

### Study (B): effect of rubbing hands during the drying procedure on the count of bacteria transferred from the surface of the skin

The performance of the Airblade™ dryer was compared with that of the A5 and Turbodry™ machines – the latter two, each being evaluated with and without hand rubbing. For comparison purposes, paper towels (Hostess folded towels; Kimberly-Clark Ltd, West Malling, UK) were also included as these are a traditional and frequently used means of drying the hands. These tests did not involve deliberate contamination of the hands. They were designed specifically to assess the relative change in numbers of bacteria transferred from the palms, fingers and fingertips postwash *vs* prewash, when hands were dried with and without rubbing. Unlike Study (A), soap was not used in the washing process, because detergents can affect the break-up of skin squamae and bacterial clumps, masking effects of hand rubbing *per se*.

On six separate occasions (different days or am and pm of same day), each volunteer reported to the laboratory and washed their hands under running water (but without soap) for 60 s, in accordance with EN1499:1997. The hands were washed in tap water at a temperature of 40 ± 5°C and a constant flow rate of 4 l min^−1^. After washing, hands were shaken five times to remove excess water. Immediately after this, bacteria were sampled from the palms, fingers and fingertips of each hand, using TSA contact plates (Rodac plates prepared by Oxoid Ltd, area *c.* 10 cm^2^). One plate was used for the centre of the palm, one was pressed against the middle of the 2nd, 3rd and 4th fingers, and with a third plate, each finger in turn was gently sampled. Thus, three contact plates were used per hand per sampling time.

After this, the hands were dried in one of six ways (Table 1), as determined by a randomization table, and bacterial sampling was repeated with fresh contact plates. The drying time was set at 15 s, in accordance with Yamamoto *et al.* (2005). In the case of paper towels, two towels were used, with the hands towelled for 15 s in accordance with the volunteer’s normal procedure. Where hands were rubbed, the hand movements of the standard EN1499:1997 handwash procedure were followed.

**Table 1:** Drying procedures compared in Study (B)

Test	Dryer/type	Procedure (all 15 s duration)
1	Airblade™/ultra-rapid	Hands kept still
2	Turbodry™/warm air	Hands kept still
3	Turbodry™/warm air	Hands rubbed
4	A5/warm air	Hands kept still
5	A5/warm air	Hands rubbed
6	Paper towels	Hands rubbed

Contact plates were incubated at 37°C for 24 h, and the number of colonies counted with the aid of a magnifying lens. Reductions in colony counts per sampling site were then calculated for the various drying procedures.

### Statistical analysis

Experimental data were analysed using the Students *t*-test function in Microsoft Excel, with a confidence interval of 95%. A value of *P* < 0·05 was taken to denote statistical significance.

## Results

### Study (A): quantification of bacteria transferred to an inert surface from the fingertips after drying

**Figure 2:**
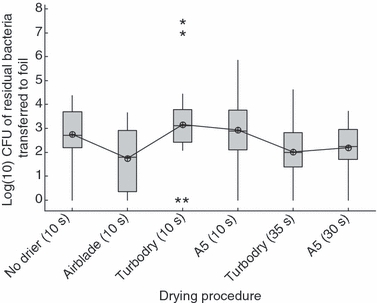
Box plot showing counts of bacteria transferred onto foil by the fingertips of each hand during tests with the different dryers in Study (A). (outliers are indicated by *).

In these tests, volunteers handled raw chicken, and after a standard handwash and use of one of the different dryers, the transfer of residual bacteria from the fingertips to foil was quantified. The results are summarized in the box plot in Fig. 2. The counts on the vertical axis represent combined transfer from all five fingers of each hand. The statistical significance of the results, together with mean log bacterial counts achieved, and standard deviations are summarized in Table 2.

**Table 2:** Mean log bacterial counts, standard deviations, and *P*-values for tests carried out in Study (A). Statistically significant results are marked with shading. Analysis includes the outliers indicated in Fig. 2

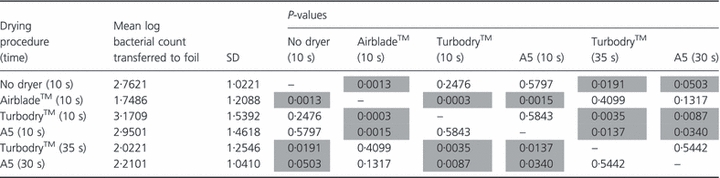

Despite undertaking a thorough handwash with soap and running water, transfers of up to 10^7^ CFU per five fingers were observed. There were 21 instances of no bacteria being transferred from a hand, and this occurred most often (seven instances) with the Airblade™, followed by the Turbodry™ (five instances, when used for 35 s). Comparing the results in Fig. 2 with those in Table 2, it can be seen that when a standard drying time of 10 s was applied, the Airblade™ unit performed considerably better (i.e. resulted in less residual bacterial transfer) than all the other methods of drying, with all the results being strongly statistically significant (i.e. *P* < 0·050). When the manufacturer’s preset device activation times were used with the Turbodry™ and A5 machines, their performance greatly improved (*P* < 0·050), but was still less than that observed with the Airblade™ unit after just 10 s. For the drying time of 10 s, both the Turbodry™ and A5 machines were associated with higher mean levels of bacterial transfer than when using no dryer at all, but the results were not statistically significant (*P* > 0·050).

**Figure 3:**
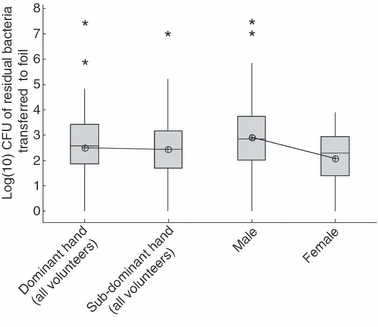
Box plot of dominant *vs* subdominant hand, and male *vs* female data acquired from all 14 volunteers for the residual bacterial transfer tests carried out in Study (A). (outliers are indicated by*).

Figure 3 is a box plot summary of the collective data (all drying methods) for the seven male and seven female volunteers. This indicates that on average, female volunteers transferred considerably fewer residual bacteria to the aluminium foil strips than the male volunteers, after washing their hands following the handling of raw chicken.

Figure 3 also shows the box plot summary of the collective data for the dominant *vs* subdominant hands of the volunteers. The data are presented in this way because some of the volunteers were left handed. It can be seen that when considering all volunteers together, after drying, there was no significant difference (*P* = 1·000) between bacterial transfer from the dominant *vs* subdominant hands. This demonstrates the appropriateness of using data from the left and right hands of a volunteer as replicates in the overall data analysis.

### Study (B): effect of rubbing hands during the drying procedure on the bacterial count on the surface of the skin

**Figure 4:**
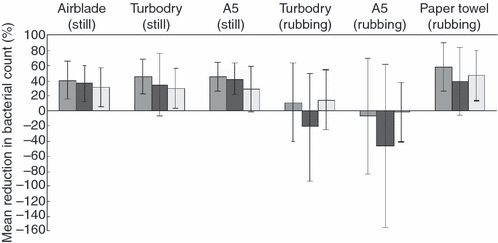
Summary of the results of Study (B): Effect of rubbing hands during the drying procedure on the bacterial count on the surface of the skin.(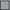) Palms; (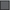) Middle of fingers; (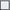) Finger tips.

The results of Study (B) are summarized in Fig. 4, which gives the mean % reduction in bacterial count achieved by the six drying procedures, after 15 s of use (Table 1). The percentage reduction in bacterial release achieved with the various drying procedures for the palms, middle of the fingers and the finger tips, respectively, are shown. Where a negative value is recorded, it means that there was an increase in the bacterial count on that area of the volunteer’s hands after the drying process, relative to the postwash sample.

These results show that rubbing of hands when using the Turbodry™ and A5 machines greatly inhibited the overall reduction in bacterial numbers released from the surface of the skin. In many instances, the bacterial numbers transferred from hands actually increased because of the rubbing action. The results obtained from the rubbing experiments were, in most cases, statistically significantly different from those obtained when holding hands still under the same devices (*P* < 0·050). When keeping the hands still, there was no statistical difference between any of the dryers, for any anatomical site, and the bacterial reduction in the middle of the fingers was comparable to that obtained with paper towel drying. Rubbing the hands with paper towels proved to be very effective at removing bacteria from the hands, with results that were in most cases statistically significant (*P* < 0·050). In particular, rubbing with paper towels appeared to be the best means of reducing bacterial loading on the fingertips.

## Discussion

Hands are washed primarily with the intention of removing transient pathogens. Substantive removal of the resident commensal flora requires the use of much more vigorous surgical-scrub type methods. The protocol for hand contamination used in Study (A) was selected because it replicates a very common scenario encountered in domestic or commercial kitchens (i.e. the handling of raw meat), which requires a person to remove transient pathogenic microbes from their hands to break the chain of transmission to another surface. The relative microbiological/hygiene risk associated with this type of contamination is emphasized by the fact that 12 (42·9%) of the 28 chickens used tested positive for the presence of one or more presumptive pathogens, such as *Campylobacter* spp., *Salmonella* spp. and *Listeria* spp. (data not shown). A diverse mixture of bacteria (transient and commensal) typically survived the handwash and was subsequently transferred to the inert foil from the volunteers’ fingers. Given that the standardized handwash lasted 60 s (i.e. longer than the period for which many people routinely wash their hands), the high levels of transients acquired from the chickens, which were still being transferred, are a cause for concern.

From the results of Study (A) (i.e. Fig. 2 and Table 2), it can be seen that residual moisture levels appear to play a critical role in determining the quantity of bacteria that are transferred from fingertips to the next items touched. This is demonstrated by the results for the Turbodry™ and A5 machines which performed much better when used for 35 and 30 s, respectively, compared with when they were used for just 10 s. For both warm air dryers, the differences associated with the two time periods were statistically significant (i.e. *P* < 0·050). Clearly, the longer the dryers were in operation, the drier the hands of the volunteers became, and thus fewer bacteria were transferred from the fingertips to the foil. The higher levels of bacterial transfer observed with the male volunteers (Fig. 3) are probably a function of the larger surface area of their fingers. Overall, the results are in agreement with those of Patrick *et al.* (1997) and Merry *et al.* (2001), both of whom found that the wetness of hands greatly influenced bacterial transfer and dissemination. If hands are not dried properly after washing, then bacterial transfer is more likely to occur. However, for most electric dryers, what constitutes an ‘adequate’ drying time is ill-defined.

The results of Study (A) suggest that if people use conventional warm air hand dryers for at least 30 s, then it is likely that the hygiene benefit will be similar to that achieved with 10 s use of the Dyson Airblade™ machine. However, if the drying time is much <30 s, the Airblade™ unit is hygienically superior for reducing transfer of microbes to other surfaces. With respect to this, the length of time people use a warm air dryer for is highly variable. Most dryers operate with a preset timer mechanism, which is generally set for about 30 s (Blackmore 1989; Redway and Knight 1998), but this is not necessarily the length of time that people keep their hands under the air stream. Redway and Knight (1998) found that men and women spent an average of 20 or 25 s, respectively, rubbing their hands in the air stream. Patrick *et al.* (1997) observed the average time for men using warm air dryers was 17 s, while for women it was just 13·3 s. In an observational study undertaken in 2006 by Dyson Limited (unpublished, data on file) in the washrooms of a motorway service station, 5000 hand drying events were timed. Men used the warm air dryers for an average of 20 s, whilst women used them for just 16 s. Interestingly, it was observed that 37% of women spent not more than 10 s attempting to dry their hands, while only 9% of women were prepared to spend 30 s or more at the dryers. Given that these reported mean drying times are substantially below 30 s, it can only be concluded that most users of warm air dryers do not achieve full drying of their hands, and thus there is greater potential for bacterial transfer from the hands and fingertips to the next surface that is touched. It should also be noted that drying hands under a warm air dryer for 30 s does not necessarily guarantee that the hands will be dry. Indeed, Redway and Knight (1998) state that for warm air dryers the average time required to achieve 95% dryness is 43 s.

Many users of warm-air hand dryers cut short the drying process simply because it takes too long and they are not prepared to wait. The shorter drying time of the Airblade™ machine may help to overcome this problem and thus improve compliance (i.e. in terms of both encouraging people to use the drying device and ensuring that the user’s hands are actually dry when they leave the unit). In theory, this could result in health benefits, as greater hand drying compliance will help reduce the spread of infectious agents by the hand-borne route.

The issue of hand rubbing was investigated in Study (B). Rubbing the hands whilst using a warm air dryer had a profound effect on aerobic bacterial counts on the surface of the skin. When hands were held stationary (palm up) in the air stream under these units, the reduction in counts of bacteria subsequently transferred from the skin was much greater than when the hands were rubbed together. Indeed, for some sites, the bacterial count increased markedly when hands were rubbed (Fig. 4). This observation correlates with the findings of Yamamoto *et al.* (2005) in similar tests. It appears that the act of briskly rubbing the hands together disturbs the outer skin squamae and brings bacteria from within the pores to the surface. Another factor, which may contribute, is the detergent action of the soap, breaking up clumps of commensal bacteria such as staphylococci and the propionibacteria, thereby increasing the number of CFU. Thus, to discount the effects of detergent and to focus on the rubbing motion, Study (B) was undertaken without the use of soap.

The ‘stationary hand’ results for the warm air machines are only for illustrative purposes. In reality, the users of such units will naturally rub their hands in the warm air stream and so are likely to increase numbers of bacteria on the surface of their hands, in contrast to those using the Airblade™ machine. The additional bacteria liberated from skin squamae or inside the pores by the rubbing action are likely to be part of the person’s normal microflora, assuming substantive removal of transients during the wash. Whilst their pathogenic potential for the host is likely to be low, they could constitute a threat if transferred to immunocompromised individuals, or those with open wounds. Drying procedures that help minimize the levels of bacteria on the skin, and colony-forming units being passed on are thus desirable in healthcare settings. Further investigations are warranted to assess what health benefits might result from their application. In relation to this, we are currently investigating whether microbes are dispersed from hand driers (including the Airblade™) and paper towels during the drying event and whether this leads to contamination of the surrounding environment.

In Study (B), the use of paper towels consistently out-performed all the other drying techniques, especially with regard to bacteria left on the palms and fingertips. This suggests that bacteria re-populating the surface of the skin during the rubbing process were being physically removed by the paper towels along with the moisture (Blackmore 1989; Redway 1994; Taylor *et al.* 2000). In so doing, paper towels appear to remove bacteria in a way in which conventional warm air dryers are incapable of replicating. However, it should be noted that towels can become highly contaminated (Taylor *et al.* 2000), something which in itself could pose a hygiene hazard. Hygienic disposal of soiled paper towels is an inherent logistical problem with this technology, especially in situations where demand for hand drying is high, such as in public washrooms. Receptacles can rapidly become full, and stocks of clean towels can become exhausted. When this happens, washed hands remain damp and the risk of bacterial transfer will increase.

The study reported here conclusively demonstrates that effective hand drying is important in preventing the postwash translocation of bacteria from the surface of hands to the next surfaces touched. The results provide an evidence base for the development and enhancement of hygienic hand drying practices. The ultra-rapid Airblade™ hand dryer was shown to be superior to the warm air dryers for reducing bacterial transfer. The lack of paper waste, coupled with its short, 10 s drying time and use of HEPA-filtered air should encourage greater compliance with hand drying and thus help reduce the spread of infectious agents by the hand-borne route.
